# The first study of reference intervals for blood biochemistry of healthy pet Pacific parrotlets (*Forpus coelestis*) in Thailand

**DOI:** 10.5455/javar.2025.l902

**Published:** 2025-04-16

**Authors:** Benchapol Lorsunyaluck, Orapat Kaewthumchai, Chaya Tinnakorn, Tanawan Phisitwanich, Sasipat Putsetkun, Natapol Pumipuntu

**Affiliations:** 1 Panalai Veterinary Hospital, Pakkret, Nonthaburi, Thailand; 2 Zoetis (Thailand) Limited Head Office, Bangrak, Bangkok, Thailand; 3 One Health Research Unit, Mahasarakham University, Maha Sarakham, Thailand; 4 Faculty of Veterinary Sciences, Mahasarakham University, Maha Sarakham, Thailand

**Keywords:** Blood biochemistry;, *Forpus coelestis*;, Pacific parrotlet;, psittacine health;, reference intervals

## Abstract

**Objective:**

This study aimed to establish baseline biochemical reference values for apparently healthy Pacific parrotlets in Thailand.

**Materials and Methods:**

Blood samples were obtained from 30 healthy individuals, and analyses were conducted using the VETSCAN^®^ VS2 with Avian/Reptilian Profile Plus rotors.

**Results:**

The results showed the measured biochemical parameters with the reference intervals included aspartate aminotransferase (73–274 U/L), glucose (235–324 mg/dl), total protein (2–3.3 gm/dl), albumin (1.5–2.5 gm/dl), globulin (0.1–1.2 gm/dl), phosphorus (1.2–5 mg/dl), calcium (7.9–9.7 mg/dl), sodium (147–157 μmol/l), potassium (2.2–3.8 μmol/l), bile acids (0–34 μmol/l), creatine kinase (44–543 U/L), uric acid (1.4–9.7 mg/dl), plasma protein (3–5 gm/dl), and packed cell volume (49%–55%).

**Conclusion:**

This study offers the first reference biochemistry values specific to Pacific parrotlets in Thailand, contributing to improved veterinary clinical practices, accurate diagnosis, and effective care for this species.

## Introduction

The Pacific parrotlet (*Forpus coelestis*), a small psittacine species, has emerged as a popular pet in Thailand, valued for its compact size, vivid plumage, and engaging behavior. As one of the smallest members of the Psittacidae family, Pacific parrotlets are ideal companions for pet owners who prefer smaller birds with the intelligence and personality of larger parrots [1]. Despite their increasing popularity, there remains a paucity of research on this species, particularly regarding standardized health assessments. Given the tendency of birds to mask signs of illness as a protective adaptation, reliable health reference intervals are essential for proactive disease monitoring and early diagnosis in veterinary practice. For Pacific parrotlets specifically, data on baseline biochemical values remain unavailable, posing challenges to clinicians in accurately diagnosing and treating health conditions in this species.

In avian medicine, biochemical reference values are indispensable for diagnosing and managing various health conditions. For psittacine species, blood biochemistry profiles can help detect liver disease, metabolic disturbances, and nutritional imbalances, which are relatively common in captive birds [2,3]. Although reference intervals have been established for larger psittacine species, including African Grey Parrots, Amazon Parrots, and Cockatoos, these values cannot be directly applied to Pacific parrotlets due to physiological differences and size-related metabolic variations [4,5]. The absence of species-specific reference intervals for biochemical markers in Pacific parrotlets limits veterinarians’ ability to provide precise, data-informed care, which is critical for species with unique physiological needs. Developing accurate biochemical baselines is thus essential for clinical decision-making, enhancing both preventive and therapeutic care for this species.

Recent technological advancements, including the development of point-of-care analyzers like the VETSCAN^®^ VS2 Chemistry Analyzer, have made it possible to conduct comprehensive biochemical analyses with small blood samples. These tools enable efficient, minimally invasive testing, which is particularly advantageous for small bird species where blood volume collection is limited [6]. Many previous studies had a VETSCAN^®^ VS2 Chemistry Analyzer to study the standard blood chemistry in many species, such as in the study of free-living loggerhead sea turtles (*Caretta caretta*) [6], the study from 12 Strigiformes species [7], the study from 80 macaw birds [8], the study from 37 American alligators [9], and the study of biochemical blood profiles in 41 common chameleons [10]. By the way, this method was very limited to small birds, including Pacific parrotlets. So, in our study, we intend to create a baseline of blood biochemistry value for healthy adult captive Pacific parrotlets for better disease. This study aims to fill the current knowledge gap by establishing blood biochemistry reference intervals specific to healthy Pacific parrotlets in Thailand. By providing a comprehensive set of baseline values, this research will support veterinarians in monitoring the health of Pacific parrotlets, contribute to the early detection of disease, and improve health management strategies in veterinary practice for this growing pet population.

## Materials and Methods

### Ethical approval

All protocols involving animal sample collection adhered to ethical guidelines, with informed consent obtained from pet owners prior to sample collection at participating animal hospitals. In addition, all of the sample collection protocol was approved by the Animal Ethics Committee of Mahasarakham University (protocol numbers: IACUC-MSU-28/2024).

### Animals

**Figure 1.**
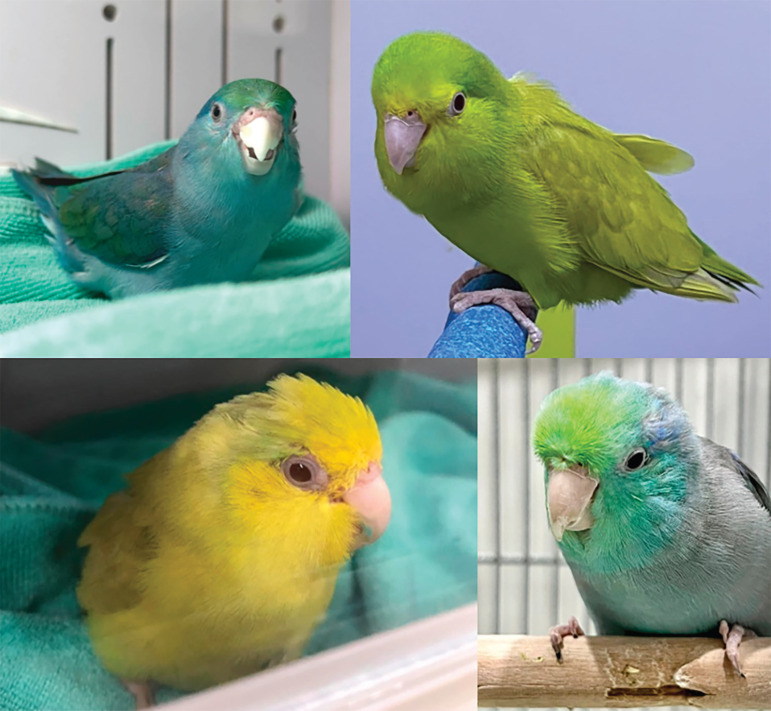
Captive pacific parrotlets (*F. coelestis*) which were assessed the blood biochemistry values at Panalai Veterinary Hospital, Nonthaburi, Thailand, and Animal Hospital, Faculty of Veterinary Sciences, Mahasarakham University, Thailand. [Photo by Benchapol Lorsunyaluck].

Thirty apparently healthy adult Pacific parrotlets (*F. coelestis*) presenting for routine health check-ups were selected for this study. These birds were evaluated at two locations in Thailand: Panalai Veterinary Hospital in Nonthaburi and the Animal Hospital, Faculty of Veterinary Sciences, Mahasarakham University. Birds included in the study exhibited normal body condition (score of 3/5), were above three months of age, had completed hand feeding, and were visibly healthy, displaying bright, alert, and responsive behaviors as shown in Figure 1 exclusion criteria included birds with abnormal droppings, reproductive activity (e.g., egg-laying), or any clinical signs of disease.

### Sample collection

Blood samples were collected via venipuncture of the right jugular vein using a 27-gauge needle and a 1-ml syringe. The blood volume obtained did not exceed 1% of each bird’s body weight to minimize the risk of hemodynamic compromise. Following collection, samples were immediately transferred to lithium heparin tubes to prevent clotting and gently mixed to ensure homogeneity.

### Hematology and blood chemistry analysis

Biochemical analysis was performed using the VETSCAN^®^ VS2 Chemistry Analyzer equipped with Avian/Reptilian Profile Plus rotors, which allows for a comprehensive metabolic panel from small sample volumes. Plasma was separated by centrifugation of whole blood at 12,000 rpm for 5 min in a Biolab^®^ TG12X microhematocrit centrifuge, with analyses conducted within 15 min post-collection to preserve sample integrity. Plasma protein concentration was determined by placing a drop of plasma on a refractometer, while packed cell volume (PCV) was measured using the microhematocrit method.

### Biochemical parameters measured

The parameters measured included aspartate aminotransferase (AST), glucose (GLU), total protein (TP), albumin (ALB), globulin (GLOB), phosphorus (PHOS), calcium (Ca²⁺), sodium (Na⁺), potassium (K⁺), bile acids (BA), creatine kinase (CK), uric acid (UA), plasma protein (PP), and PCV. Any sample displaying hemolysis, lipemia, or icterus was excluded from the analysis to ensure the accuracy of reference intervals. Additionally, data points exceeding ±3 SDs from the mean were removed to establish reliable reference values.

### Statistical analysis

Data analysis was performed using SPSS Statistics software, version 22. Descriptive statistics, including mean and SD, were calculated for each biochemical parameter. Reference intervals of blood biochemical values were established based on test values that fell within two SDs, representing 95% of a normal distribution for the sampled reference population. Test results below the 2.5th percentile and above the 97.5th percentile were excluded.

## Results

### Hematology and biochemical reference intervals

The reference intervals for hematological and biochemical parameters in healthy adult Pacific parrotlets (*F. coelestis*) were analyzed based on data from 30 individuals. Table 1 summarizes the minimum, maximum, mean (± SD), and reference range values for each parameter. Reference intervals were calculated for AST, GLU, TP, ALB, GLOB, PHOS, Ca²⁺, Na⁺, K⁺, BA, CK, UA, PP, and PCV.

**Table 1.** Clinical blood biochemistry in pacific parrotlets.

Blood biochemistry	Unit	Minimum value	Maximum value	x̄ ± S D	Reference value
AST	U/L	80	272	173.21 ± 50.20	73–274
GLU	mg/dl	225	332	279.27 ± 22.16	235–324
TP	gm/dl	2.1	3.4	2.63 ± 0.32	2–3.3
ALB	gm/dl	1.5	2.5	2.01 ± 0.26	1.5–2.5
GLOB	gm/dl	0.1	1.2	0.61 ± 0.28	0.1–1.2
PHOS	mg/dl	1.1	5.1	3.05 ± 0.95	1.2–5
Ca2+	mg/dl	8	10.1	8.82 ± 0.46	7.9–9.7
Na+	μmol/l	146	157	152.07 ± 2.5	147–157
K+	μmol/l	2.4	3.7	3.00 ± 0.39	2.2–3.8
BA	μmol/l	0	41	15.58 ± 9.29	0–34
CK	U/L	139	581	293.43 ± 124.61	44–543
UA	mg/dl	1.7	10.5	5.54 ± 2.08	1.4–9.7
PCV	%	49	55	52.00 ± 1.58	49–55
PP	gm/dl	3	5	3.97 ± 0.5	3–5

*AST* = aspartate aminotransferase, *GLU* = glucose, *TP* = total protein, *ALB* = albumin, *GLOB* = globulin, *PHOS* = phosphorus, *Ca²⁺* = calcium, *Na*⁺ = sodium, *K*⁺ = potassium, *BA* = bile acids, *CK* = creatine kinase, *UA* = uric acid, *PP* = plasma protein, *PCV* = packed cell volume.

### Biochemical parameter analysis

The biochemical analysis revealed that AST levels ranged from 73 to 274 U/L, with a mean of 173.21 ± 50.20 U/L. GLU concentrations were recorded between 235 and 324 mg/dl (mean: 279.27 ± 22.16 mg/dl). Protein values showed a TP range of 2.0 to 3.3 gm/dl, ALB levels from 1.5 to 2.5 gm/dl, and GLOB levels between 0.1 and 1.2 gm/dl. The PHOS levels varied from 1.2 to 5.0 mg/dl, while Ca^2^^+^ levels were measured between 7.9 and 9.7 mg/dl. Electrolytes, including Na^+^ and K^+^, ranged from 147 to 157 μmol/l and 2.2 to 3.8 μmol/l, respectively. BA concentrations varied from 0 to 34 μmol/l. CK values were broad, ranging from 44 to 543 U/L, while UA was between 1.4 and 9.7 mg/dl. PP and PCV showed ranges of 3.0 to 5.0 gm/dl and 49 to 55%, respectively. These findings can help in establishing the first reference intervals for these biochemical markers in Pacific parrotlets (*F. coelestis*).

### Exclusion of outliers

Certain data points were excluded from the final reference ranges to ensure data accuracy. Specifically, samples with values exceeding ±3 SDs from the average for AST, Ca^2^^+^, BA, CK, UA, PCV, and PP were discarded. Additionally, BA and CK readings that could not be analyzed by the VETSCAN^®^ VS2 were marked as zero and excluded to prevent skewed reference intervals. All other samples were within acceptable ranges, with no evidence of hemolysis, lipemia, or icterus in the analyzed blood samples.

## Discussion

This study presents the first established reference intervals for blood biochemical parameters in healthy Pacific parrotlets (*F. coelestis*) in Thailand. These intervals provide critical baseline data for veterinarians in the early detection of diseases, health monitoring, and management of this increasingly popular pet species. The values obtained in our study, including key parameters like AST, GLU, and TP, offer a foundational tool for clinical assessments, enabling accurate differentiation between healthy and pathological states in Pacific parrotlets [11–14]. Given the limited availability of species-specific reference data for smaller psittacine birds, these findings bridge a crucial gap in avian clinical care.

A significant observation in our study was the broad range in CK values among sampled Pacific parrotlets. Elevated CK levels have been reported in association with muscle activity and stress-related responses in avian species, which may contribute to the variability seen here [15,16]. Factors such as handling stress, flight, or minor trauma during capture and restraint could stimulate CK secretion, as CK plays a role in ATP production required for muscle contraction [17]. Additionally, blood sampling methods, such as incomplete tube sealing, may influence CK levels by allowing changes in pH due to carbon dioxide leakage [3,18]. This variability suggests that CK levels should be interpreted with caution in clinical settings, accounting for recent physical activity and handling history.

BA are valuable indicators of hepatic function in birds; however, our study found BA levels that were broad yet largely below 35 μmol/l, as measured by the VETSCAN^®^ VS2 analyzer. This range may reflect the analyzer’s limitations or physiological variations within this species [6]. Similarly, PCV values in the studied Pacific parrotlets ranged from 49% to 55%, providing a valuable reference for hydration status and anemia assessment in clinical evaluations. Notably, PP values were consistently higher than TP values, likely due to the presence of additional proteins involved in inflammatory responses, as refractometry may also capture these proteins [19,20].

Our reference intervals show both similarities and differences when compared to larger psittacine species. For instance, AST and GLU values in Pacific parrotlets align with ranges seen in Amazon and African Grey parrots, suggesting some metabolic consistency across psittacine species [5,8]. However, variations in electrolyte levels, such as K⁺ and Na⁺, may reflect species-specific metabolic needs or dietary influences unique to Pacific parrotlets [21,22]. Such interspecies differences underscore the importance of establishing species-specific reference intervals rather than relying on general psittacine data, as extrapolations can lead to inaccurate diagnoses and inappropriate management [23–25].

The biochemical reference intervals established in this study are critical for veterinarians in avian clinical management as well as assessing Pacific parrotlet health [26–30]. By the way, limitations must be acknowledged. Environmental and management factors, such as diet, exercise, and housing conditions, can affect baseline biochemical levels and were not fully controlled in this study. Future studies incorporating larger sample sizes, including both captive and wild populations, would help validate and refine these findings. Additionally, technical variability associated with diagnostic equipment, such as refractometry for protein measurements, may influence results and should be standardized across clinics to improve diagnostic consistency.

## Conclusion

To the best of our knowledge, this study provides the first comprehensive set of biochemical reference intervals for Pacific parrotlets in Thailand, offering a crucial diagnostic resource for veterinary practitioners. These intervals enable more precise health assessments, improving disease detection, clinical care, and preventive health strategies for this popular psittacine species. Future research focusing on population-based studies and broader geographic sampling is recommended to further establish and validate these baseline values, enhancing the veterinary community’s capacity to support Pacific parrotlet health and welfare.

## List of abbreviations

AST, aspartate aminotransferase; ALB, albumin; BA, bile acids; Ca²⁺, calcium; CK, creatine kinase; GLU, glucose; GLOB, globulin; K⁺, potassium; Na⁺, sodium; UA, uric acid; PHOS, phosphorus; PP, plasma protein; PCV, packed cell volume; TP, total protein.

## References

[1] Bocalini F, Silveira LF (2015). Morphological variability and taxonomy of the blue-winged parrotlet *Forpus xanthopterygius* (*Psittacidae*). Rev Bras Ornitol.

[2] Polo FJ, Peinado VI, Viscor G, Palomeque J (1998). Hematologic and plasma chemistry values in captive psittacine birds. Avian Dis.

[3] Campbell TW, Ellis CK (2015). Avian and exotic animal hematology and cytology.

[4] Pinto FE, Andrade TU, Endringer DC, Lenz D (2016). Hematological and serum biochemical reference values for captive blue-fronted amazon parrots. Comp Clin Path.

[5] Di Santo LG, Braos LB, Kawanami AE, Oliveira JP, Cruz ND, Mendonça FS (2018). Feed processing effects on digestibility, palatability, excreta fermentation products, and blood parameters in blue-fronted amazon parrots (*Amazona aestiva*). J Anim Physiol Anim Nutr.

[6] Atkins A, Jacobson E, Hernandez J, Bolten AB, Lu X (2010). Use of a portable point-of-care (Vetscan VS2) biochemical analyzer for measuring plasma biochemical levels in free-living loggerhead sea turtles (*Caretta caretta)*. J Zoo Wildl Med.

[7] Ammersbach M, Beaufrère H, Gionet Rollick A, Tully T (2015). Laboratory blood analysis in Strigiformes-Part I: Hematologic reference intervals and agreement between manual blood cell counting techniques. Vet Clin Pathol.

[8] Peruffo L, Boyd JD, Hoppes S, Brightsmith DJ (2016). Blood biochemical values of wild scarlet macaw (*Ara macao macao*) nestlings and adults. J Avian Med Surg.

[9] Hamilton MT, Kupar CA, Kelley MD, Finger JWJ, Tuberville TD (2016). Blood and plasma biochemistry reference intervals for wild juvenile American alligators (*Alligator mississippiensis*). J Wildl Dis.

[10] Eshar D, Ammersbach M, Shacham B, Katzir G, Beaufrère H (2018). Venous blood gases, plasma biochemistry, and hematology of wild-caught common chameleons (*Chamaeleo chamaeleon*). Can J Vet Res.

[11] Gallo L, Quintana F, Svagelj WS, Uhart M (2013). Hematology and blood chemistry values in free-living imperial cormorants (*Phalacrocorax atriceps*). Avian Dis.

[12] Pavel O, Abbas B, Dyary H (2022). Some physiological and serum biochemical reference values of adult chukar partridge (*Alectoris chukar Kurdestanica*) in Kurdistan region-Iraq. Al-Anbar J Vet Sci.

[13] Chaprazov T, Petrov R, Yarkov D, Andonova Y, Lazarova I (2023). Basic blood biochemical parameters of wild common ravens (*Corvuscorax*). Biodivers Data J.

[14] Carrillo-Muro O, Rodríguez-Cordero D, Hernández-Briano P, Correa-Aguado PI, Medina-Flores CA, Huerta-López LA (2024). Enzymic activity, metabolites, and hematological responses in high-risk newly received calves for “clinical health” reference intervals. Animals.

[15] Guglielmo CG, Piersma T, Williams TD (2001). A sport-physiological perspective on bird migration: Evidence for flight-induced muscle damage. J Exp Biol.

[16] Baird MF, Graham SM, Baker JS, Bickerstaff GF (2012). Creatine kinase-and exercise-related muscle damage: Implications for muscle performance and recovery. J Nutr Metab.

[17] Hettling H, Van Beek JH (2011). Analyzing the functional properties of the creatine kinase system with multiscale ‘sloppy’ modeling. PLoS Comput Biol.

[18] Lippi G, Avanzini P, Cosmai M, Aloe R, Ernst D (2012). Incomplete filling of lithium heparin tubes affects the activity of creatine kinase and gamma-glutamyltransferase. Br J Biomed Sci.

[19] Chitty J (2018). Sample taking and basic clinical pathology. BSAVA Manual Avian Pract.

[20] Kaneko JJ, Harvey JW, Bruss ML (2019). Clinical biochemistry of domestic animals.

[21] Qian Q (2018). Dietary influence on body fluid acid-base and volume balance: the deleterious “norm” furthers and cloaks subclinical pathophysiology. Nutrients.

[22] Morris AL, Mohiuddin SS (2023). Biochemistry, nutrients.

[23] Hung CS, Sladakovic I, Divers SJ (2020). Diagnostic value of plasma biochemistry, haematology, radiography and endoscopic visualisation for hepatic disease in psittacine birds. Vet Rec.

[24] White SD, Beaufrère H, Guzman DS, Affolter VK, Tell LA, Paul-Murphy J (2025). Cutaneous disorders in captive psittacines, a retrospective study of 1454 cases at a university veterinary teaching hospital (1988–2021). Vet Dermatol.

[25] Thrall MA, Baker DC, Campbell TW (2022). Veterinary hematology, clinical chemistry, and cytology.

[26] Karki B, Lamichhane BR, Sadaula A, Khadka BB, Bhusal KP (2020). Hematological study of captive white-rumped vultures (*Gyps bengalensis*) to assess their health status. J Avian Med Surg.

[27] Kim M, Wut Hmohn ZZ, Jang W, Baek G, Han JI (2025). Hematologic and clinical chemistry reference intervals for six species of wild birds frequently rescued in the Republic of Korea. Front Vet Sci.

[28] Gaspar H, Bargallo F, Grifols J, Correia E, Pinto M (2021). Age and sex-related differences in the haematological parameters of captive African grey parrots (*Psittacus erithacus*). Vet Med-Czech.

[29] Nwaigwe CU, Ihedioha JI, Shoyinka SV, Nwaigwe CO (2020). Evaluation of the hematological and clinical biochemical markers of stress in broiler chickens. Vet World.

[30] Kordestani H, Abdi-Hachesoo B, Bakhshaei F, Safaeian S, Nazifi S (2022). Hematological and serum biochemical parameters of the captive long-legged buzzard (*Buteo rufinus*) in Iran. Vet Med Sci.

